# Comparison of Insomnia Risk between Spironolactone and Esaxerenone in Individuals with Hypertension

**DOI:** 10.31662/jmaj.2025-0554

**Published:** 2026-05-29

**Authors:** Takashin Nakayama, Hidehiro Kaneko, Yuta Suzuki, Akira Okada, Hiroyuki Morita, Katsuhito Fujiu, Norifumi Takeda, Tatsuhiko Azegami, Takashi Yokoo, Norihiko Takeda, Koichi Node, Hideo Yasunaga, Kaori Hayashi

**Affiliations:** 1 Division of Endocrinology, Metabolism, and Nephrology, Department of Internal Medicine, Keio University School of Medicine, Tokyo, Japan; 2 Department of Cardiovascular Medicine, The University of Tokyo, Tokyo, Japan; 3 Department of Advanced Cardiology, The University of Tokyo, Tokyo, Japan; 4 Center for Outcomes Research and Economic Evaluation for Health, National Institute of Public Health, Saitama, Japan; 5 Department of Prevention of Diabetes and Lifestyle-Related Diseases, Graduate School of Medicine, The University of Tokyo, Tokyo, Japan; 6 International University of Health and Welfare, Tokyo, Japan; 7 Division of Nephrology and Hypertension, Department of Internal Medicine, Jikei University School of Medicine, Tokyo, Japan; 8 Department of Cardiovascular Medicine, Saga University, Saga, Japan; 9 Department of Clinical Epidemiology and Health Economics, School of Public Health, The University of Tokyo, Tokyo, Japan

**Keywords:** mineralocorticoid receptor antagonist, spironolactone, esaxerenone, insomnia

## Abstract

**Introduction:**

Spironolactone (SPL) is a steroidal mineralocorticoid receptor antagonist (MRA) that binds non-specifically to sex hormone receptors. Given the role of sex hormones in sleep regulation, SPL may pose a theoretical risk of insomnia through off-target activity. Recently introduced, esaxerenone (ESAX) is a non-steroidal MRA with high mineralocorticoid receptor selectivity, attracting attention for its potentially favorable safety profile.

**Methods:**

This study was conducted to compare the risk of insomnia between SPL and ESAX. Individuals with a history of hypertension who newly initiated either agent were enrolled from the DeSC database. Inverse probability of treatment weighting was used to assess differences in insomnia incidence between treatment groups.

**Results:**

We analyzed 5,091 individuals without a history of insomnia: 1,910 received SPL and 3,181 received ESAX. The median age was 72 (interquartile range [IQR], 67-79) years, 55% of participants were male, and the median estimated glomerular filtration rate was 66.3 (IQR, 56.1-76.2) mL/min/1.73 m^2^. Over a median follow-up of 512 (IQR, 240-848) days, 356 insomnia events were observed. Weighted Cox regression analysis indicated a significantly higher risk of incident insomnia among SPL users than ESAX users (hazard ratio [HR], 1.66; 95% confidence interval [CI], 1.32-2.10). In sex-stratified analyses, the HRs were 1.79 (95% CI, 1.30-2.47) for men and 1.59 (95% CI, 1.15-2.20) for women.

**Conclusions:**

Using a nationwide claims database, we demonstrated that, compared with ESAX, SPL use was associated with a greater likelihood of developing insomnia.

## Introduction

Mineralocorticoid receptor antagonists (MRAs) block aldosterone binding to mineralocorticoid receptors in the cortical collecting ducts, promoting natriuresis and lowering blood pressure ^(1), (2), (3), (4)^. Supported by evidence of organ protection, MRAs are considered one of the key antihypertensive medications in contemporary hypertension management ^(2), (3), (4), (5), (6), (7), (8)^.

Spironolactone (SPL) is a longstanding MRA approved in the 1960s ^(9), (10)^. However, due to its steroidal structure, SPL also non-selectively binds to sex hormone receptors ^(5), (11)^. It is worth noting that the link between hormonal disturbances and sleep disorders has become an area of growing interest ^(12), (13), (14), (15), (16), (17), (18)^. Insomnia, a potential bidirectional comorbidity of hypertension, is associated with adverse outcomes ^(19), (20), (21)^. Accordingly, the putative insomnia risk of SPL represents an important topic for investigation.

In recent years, non-steroidal MRAs with improved receptor selectivity have been developed, offering the prospect of reducing off-target side effects ^(11)^. Currently, two agents from this class―esaxerenone (ESAX) and finerenone―are available in Japan, though the latter has limited antihypertensive efficacy and is not approved for hypertension in Japan or in the United States ^(6), (7), (22), (23)^. Based on these considerations, this study aimed to compare the incidence of insomnia between SPL and ESAX in individuals with hypertension. As accumulating clinical evidence continues to underscore the importance of MRAs in routine practice, we believe that pharmacological epidemiologic evidence on adverse events―such as insomnia, which is both common and clinically important―is also essential for advancing hypertension management.

## Materials and Methods

### Study design

This retrospective cohort study employed a new-user, active-comparator design to compare the incidence of insomnia between SPL and ESAX ^(24)^. Data were obtained from the DeSC database (DeSC Healthcare Inc., Tokyo, Japan), a large-scale administrative claims database with annual health checkup data from April 2014 to August 2024 ^(25)^. The DeSC database integrated administrative data sourced from Japan’s three health insurers: employment-based health insurance managed by associations or unions for salaried workers in large corporations (*Kenpo*); the National Health Insurance program for non-employed individuals under the age of 75 (*Kokuho*); and the Advanced Elderly Medical Service System for those aged 75 years and older (*Koki Koreisha Iryo Seido*). Diagnoses were classified using the International Classification of Diseases, 10th Revision (ICD-10) or the Japanese-specific disease coding system, and medications were classified using the World Health Organization Anatomical Therapeutic Chemical classification or the Japanese-specific drug coding system. In Japan, most residents are encouraged to undergo an annual health checkup that includes anthropometric measurements, lifestyle questionnaires, and laboratory tests.

Figure 1 presents the flowchart of participant selection. In Japan, ESAX―unlike SPL―is contraindicated in individuals with an estimated glomerular filtration rate (eGFR) <30 mL/min/1.73 m^2^ and is approved exclusively for the treatment of hypertension, with no insurance coverage for heart failure or hepatic edema. We identified 7,814 individuals with hypertension who, to ensure comparability, met all of the following inclusion criteria: initiation of either SPL or ESAX at least 12 months after insurance enrollment, on or after May 13, 2019 (the launch date of ESAX); no history of heart failure, liver disease (liver failure, cirrhosis, or hepatic fibrosis), or kidney replacement therapy; no concomitant use of loop diuretics; and eGFR ≥30 mL/min/1.73 m^2^. Subsequently, we excluded 13 individuals with a history of gynecomastia, 2,359 with a history of insomnia, 348 who had been prescribed eplerenone or finerenone prior to the initiation of SPL or ESAX, and three who received both SPL and ESAX concurrently. The final analytic cohort comprised 5,091 individuals.

**Figure 1.**
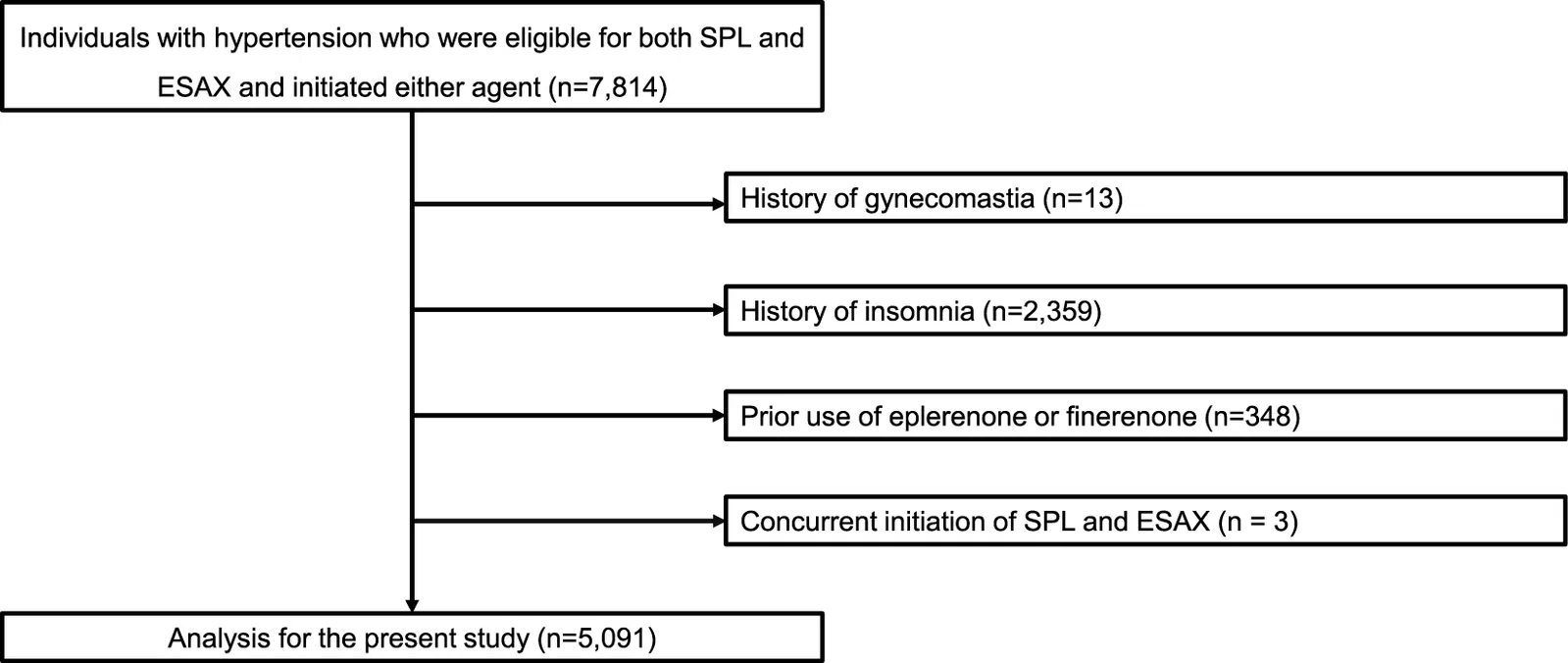
Flowchart of participant selection. Given the differences in approved indications and contraindications between SPL and ESAX, we included 7,814 individuals with a history of hypertension who initiated SPL or ESAX at least 12 months after insurance enrollment, had no history of heart failure, liver disease (liver failure, cirrhosis, or hepatic fibrosis), or kidney replacement therapy, did not use loop diuretics concomitantly, and had eGFR ≥30 mL/min/1.73 m^2^. We then excluded individuals with a history of gynecomastia (n = 13) or insomnia (n = 2,359), those prescribed eplerenone or finerenone prior to SPL or ESAX initiation (n = 348), and those who received both SPL and ESAX concurrently (n = 3). Ultimately, 5,091 individuals were included in the final analysis. eGFR: estimated glomerular filtration rate; ESAX: esaxerenone; SPL: spironolactone.

### Ethics statement

The Ethics Committee of the University of Tokyo reviewed and approved the study protocol (approval number: 2021010NI), in line with the Declaration of Helsinki. Since the DeSC database contains only anonymized data, the requirement for informed consent was waived.

### Measurements and definitions

Baseline data before SPL or ESAX initiation were obtained from health checkup records, including body mass index, blood pressure, lifestyle factors (smoking status [current or non-current] and alcohol consumption [daily or non-daily]), and laboratory results (triglycerides, high-density lipoprotein cholesterol, low-density lipoprotein cholesterol, hemoglobin A1c, and creatinine). eGFR was estimated using the equation: 194×Creatinine^-1.094^×Age^-0.287^ (× 0.739 if female) ^(26)^. Information on comorbidities and concurrent medications at the index date was retrieved from administrative claims data. In addition, diabetes mellitus was defined as hemoglobin A1c ≥6.5% or use of glucose-lowering medications, and dyslipidemia as triglycerides ≥150 mg/dL, high-density lipoprotein cholesterol <40 mg/dL, low-density lipoprotein cholesterol ≥140 mg/dL, or use of lipid-lowering agents.

### Study outcomes

The primary outcome was the incidence of insomnia, defined by ICD-10 codes F510 and G470. Participants were followed until the incidence of insomnia, insurance disenrollment, death, or the end of the study period.

### Inverse probability of treatment weighting

To account for differences in treatment assignment, stabilized inverse probability of treatment weighting (IPTW) based on the propensity score was employed ^(27)^. Propensity scores for SPL initiation were estimated using logistic regression incorporating the following covariates: age, sex, MRA initiation period (2019-2021 vs 2022-2024), smoking status, alcohol consumption, body mass index, systolic blood pressure, comorbidities (diabetes mellitus, dyslipidemia, primary aldosteronism, myocardial infarction, stroke, malignancy, depression, schizophrenia, Alzheimer’s disease, thyroid gland disorders, gastroesophageal reflux disease, sleep apnea syndrome, and overactive bladder), concurrent medications (calcium channel blockers [CCBs], renin-angiotensin system [RAS] inhibitors, thiazide-type diuretics, beta-blockers, sodium-glucose cotransporter 2 [SGLT2] inhibitors, anxiolytics, analgesics, systemic glucocorticoids, and topical glucocorticoids), and eGFR. Baseline characteristics between SPL and ESAX users were compared before and after IPTW adjustment using standardized mean differences, where absolute values <0.1 were considered adequately balanced. The association between SPL use and insomnia was evaluated using IPTW-weighted cohorts with ESAX as the comparator.

### Statistical analysis

Continuous variables were summarized as medians with interquartile ranges (IQR), and categorical variables as counts and proportions. Cox proportional hazards models were used to estimate hazard ratios (HRs) and 95% confidence intervals (CIs). Subgroup analyses were stratified by age (<75 or ≥75 years), sex, body mass index (<23 or ≥23 kg/m^2^), systolic blood pressure (<140 or ≥140 mmHg), and eGFR (<60 or ≥60 mL/min/1.73 m^2^).

We conducted six sensitivity analyses. First, we applied overlap weighting methods to achieve balance in baseline characteristics ^(28)^. Second, we included the urban status of the insurer’s location as an additional covariate to address potential regional variation in treatment allocation. Individuals for whom this information was unavailable were excluded from this analysis. Third, MRAs are often prescribed for resistant hypertension; therefore, we restricted the analysis to individuals concurrently receiving CCBs, RAS inhibitors, or thiazide-type diuretics. Fourth, we conducted an analysis including only those who received a follow-up prescription for SPL or ESAX between three and six months after initiation. Fifth, we redefined the outcome from insomnia to sleep disorders (ICD-10 codes: F51 and G47). For this analysis, individuals with a history of sleep disorders were additionally excluded. Sixth, we handled missing data on smoking status and alcohol consumption using multiple imputation. In each imputed dataset, IPTW was estimated based on propensity scores, and the resulting HRs were combined using Rubin’s rules ^(29)^.

## Results

### Participants’ backgrounds

Table 1 presents the baseline characteristics of 5,091 eligible participants, of whom 1,910 initiated SPL and 3,181 ESAX. The median age was 72 (IQR, 67-79) years, and the proportion of males was 55%. The median systolic and diastolic blood pressure was 140 (IQR, 128-154) and 79 (IQR, 70-88) mmHg, respectively. Concomitant use of CCBs, RAS inhibitors, and thiazide-type diuretics was observed in 62%, 77%, and 22% of participants, respectively. The median eGFR was 66.3 (IQR, 56.1-76.2) mL/min/1.73 m^2^. Before weighting, SPL users were older, less likely to be male, and had a higher prevalence of comorbidities, including myocardial infarction and malignancy. RAS and SGLT2 inhibitors were less prevalent in the SPL group, whereas other medications tended to be more frequently prescribed. There was no notable difference in eGFR between the two groups. After applying IPTW, all baseline covariates were adequately balanced between SPL and ESAX users (Table S1).

**Table 1.** Baseline Characteristics of the Unweighted Study Population.

	Overall (n = 5,091)	ESAX users (n = 3,181)	SPL users (n = 1,910)	Absolute SMD
Age, years	72 (67-79)	71 (65-78)	75 (69-81)	0.345
Male	2,820 (55%)	1,912 (60%)	908 (48%)	0.254
Year of MRA initiation				
2019-2021	2,634 (52%)	1,558 (49%)	1,076 (56%)	0.148
2022-2024	2,457 (48%)	1,623 (51%)	834 (44%)	0.148
Cigarette smoking				
Non-current	3,071 (60%)	1,941 (61%)	1,130 (59%)	0.038
Current	606 (12%)	413 (13%)	193 (10%)	0.090
Unknown	1,414 (28%)	827 (26%)	587 (31%)	0.105
Alcohol consumption				
Non-daily	2,268 (45%)	1,395 (44%)	873 (46%)	0.037
Daily	1,168 (23%)	818 (26%)	350 (18%)	0.179
Unknown	1,655 (33%)	968 (30%)	687 (36%)	0.118
Body mass index, kg/m^2^	24.3 (22.0-26.9)	24.5 (22.3-27.1)	23.9 (21.5-26.6)	0.144
Systolic blood pressure, mmHg	140 (128-154)	141 (130-156)	138 (127-150)	0.171
Diastolic blood pressure, mmHg	79 (70-88)	80 (70-89)	78 (70-86)	0.178
Comorbidities				
Diabetes mellitus	1,100 (22%)	704 (22%)	396 (21%)	0.034
Dyslipidemia	3,468 (68%)	2,200 (69%)	1,268 (66%)	0.059
Primary aldosteronism	168 (3%)	103 (3%)	65 (3%)	0.009
Myocardial infarction	111 (2%)	28 (1%)	83 (4%)	0.218
Stroke	545 (11%)	282 (9%)	263 (14%)	0.155
Malignancy	741 (15%)	360 (11%)	381 (20%)	0.239
Gastroesophageal reflux disease	1,973 (39%)	1,170 (37%)	803 (42%)	0.108
Thyroid gland disorders	412 (8%)	234 (7%)	178 (9%)	0.071
Sleep apnea	168 (3%)	123 (4%)	45 (2%)	0.087
Overactive bladder	597 (12%)	326 (10%)	271 (14%)	0.121
Depressive disorder	200 (4%)	111 (3%)	89 (5%)	0.059
Alzheimer’s disease	143 (3%)	64 (2%)	79 (4%)	0.123
Schizophrenia	55 (1%)	23 (1%)	32 (2%)	0.088
Medications				
Calcium channel blockers	3,147 (62%)	1,904 (60%)	1,243 (65%)	0.108
RAS inhibitors	3,900 (77%)	2,638 (83%)	1,262 (66%)	0.394
Thiazide-type diuretics	1,117 (22%)	715 (22%)	402 (21%)	0.035
Beta blockers	817 (16%)	464 (15%)	353 (18%)	0.105
SGLT2 inhibitors	361 (7%)	257 (8%)	104 (5%)	0.105
Systemic glucocorticoids	512 (10%)	213 (7%)	299 (16%)	0.287
Topical glucocorticoids	767 (15%)	424 (13%)	343 (18%)	0.128
Anxiolytics	348 (7%)	182 (6%)	166 (9%)	0.115
Analgesics	1,138 (22%)	567 (18%)	571 (30%)	0.286
Laboratory data				
LDL-C, mg/dL	115 (95-135)	115 (96-135)	115 (93-135)	0.026
HDL-C, mg/dL	59 (49-71)	59 (49-70)	59 (49-71)	0.061
Triglycerides, mg/dL	106 (77-153)	110 (79-158)	101 (74-145)	0.116
Hemoglobin A1c, %	5.7 (5.5-6.1)	5.7 (5.5-6.1)	5.7 (5.4-6.1)	0.016
eGFR, mL/min/1.73 m^2^	66.3 (56.1-76.2)	66.8 (56.5-76.3)	65.8 (55.3-76.0)	0.055

eGFR: estimated glomerular filtration rate; ESAX: esaxerenone; HDL-C: high-density lipoprotein cholesterol; LDL-C: low-density lipoprotein cholesterol; MRA: mineralocorticoid receptor antagonist; RAS: renin-angiotensin system; SGLT2: sodium-glucose cotransporter 2; SMD: standardized mean difference; SPL: spironolactone.

### Association between SPL use and insomnia onset

During a median follow-up of 512 (IQR, 240-848) days, a total of 356 insomnia events were documented. The overall incidence rate was 4.36 (95% CI, 3.93-4.84) per 100 person-years, with rates of 6.47 (95% CI, 5.65-7.41) and 3.00 (95% CI, 2.55-3.52) per 100 person-years in the SPL and ESAX groups, respectively (Table 2). In the Cox proportional hazards model adjusted using IPTW, initiation of SPL was significantly associated with a higher risk of developing insomnia compared with ESAX (HR, 1.66; 95% CI, 1.32-2.10). All subgroup analyses stratified by age, sex, body mass index, systolic blood pressure, and eGFR showed generally consistent patterns, indicating an increased likelihood of insomnia associated with SPL use (Figure 2).

**Table 2.** Risk of Insomnia Disorders among SPL and ESAX Users: Inverse Probability of Treatment Weighting Analysis.

	No. of participants	No. of events	IR (95%CI)	Crude HR (95%CI)	Weighted HR (95%CI)
SPL users	1,910	207	6.47 (5.65-7.41)	2.18 (1.77-2.70)	1.66 (1.32-2.10)
ESAX users	3,181	149	3.00 (2.55-3.52)	1 [reference]	1 [reference]

IRs were calculated per 100 person-years. Weighted HRs were estimated using Cox proportional hazards models with inverse probability of treatment weighting. The models accounted for age, sex, mineralocorticoid receptor antagonist initiation period, smoking status, alcohol consumption, body mass index, systolic blood pressure, diabetes mellitus, dyslipidemia, primary aldosteronism, myocardial infarction, stroke, malignancy, depression, Alzheimer’s disease, schizophrenia, overactive bladder, sleep apnea syndrome, gastroesophageal reflux disease, thyroid gland disorders, use of calcium channel blockers, renin-angiotensin system inhibitors, thiazide-type diuretics, beta-blockers, sodium-glucose cotransporter 2 inhibitors, anxiolytics, analgesics, systemic glucocorticoids, and topical glucocorticoids, and estimated glomerular filtration rate. CI: confidence interval; ESAX: esaxerenone; HR: hazard ratio; IR: incidence rate; SPL: spironolactone.

**Figure 2.**
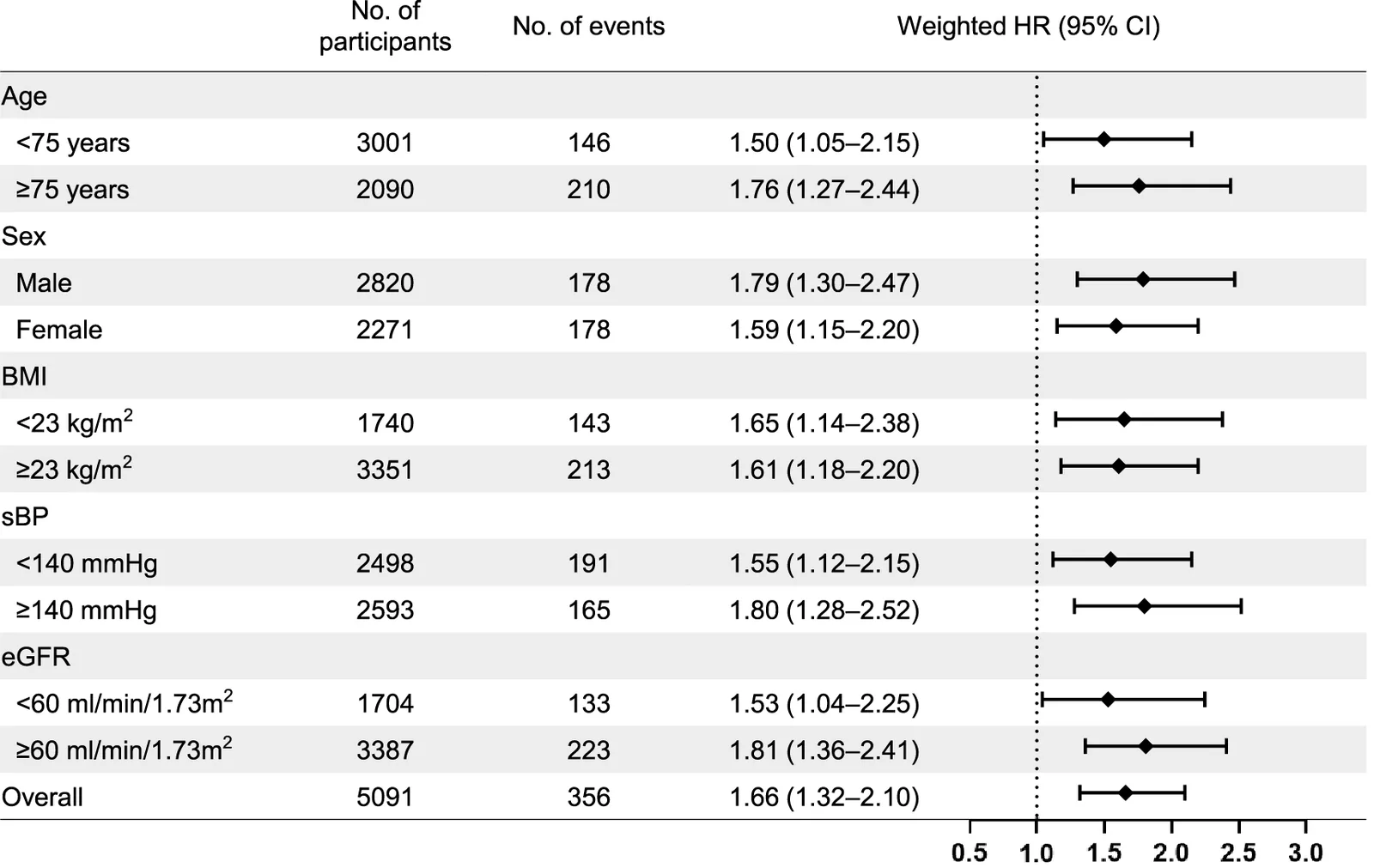
Subgroup analyses of insomnia risk with SPL vs ESAX using inverse probability of treatment weighting. We performed Cox proportional hazards regression to estimate the HR and 95% CI for the incidence of insomnia associated with SPL use compared with ESAX use, applying IPTW based on subgroup-specific propensity scores. The models were adjusted for age, sex, mineralocorticoid receptor antagonist initiation period, smoking status, alcohol consumption, BMI, sBP, diabetes mellitus, dyslipidemia, primary aldosteronism, myocardial infarction, stroke, malignancy, depression, Alzheimer’s disease, schizophrenia, overactive bladder, sleep apnea syndrome, gastroesophageal reflux disease, thyroid gland disorders, use of calcium channel blockers, renin-angiotensin system inhibitors, thiazide-type diuretics, beta-blockers, sodium-glucose cotransporter 2 inhibitors, anxiolytics, analgesics, systemic glucocorticoids, and topical glucocorticoids, and eGFR. Subgroup analyses were conducted based on age (<75 or ≥75 years), sex, BMI (<23 or ≥23 kg/m^2^), sBP (<140 or ≥140 mmHg), and eGFR (<60 or ≥60 mL/min/1.73 m^2^). In the sex-stratified analyses, sex was not included as a covariate in the propensity score estimation. BMI: body mass index; CI: confidence interval; eGFR: estimated glomerular filtration rate; ESAX: esaxerenone; HR: hazard ratio; sBP: systolic blood pressure; SPL: spironolactone.

### Sensitivity analyses

First, the overlap weighting analysis found that SPL use was significantly associated with an increased risk of insomnia compared with ESAX use (HR, 1.77; 95% CI, 1.41-2.23) (Table S2). Second, among 4,624 participants with available urbanicity data, SPL use was a predictor of insomnia onset (HR, 1.64; 95% CI, 1.29-2.09) (Table S3). Third, after excluding 375 participants not concurrently taking CCBs, RAS inhibitors, or thiazide-type diuretics, our main findings remained unchanged (HR, 1.62; 95% CI, 1.28-2.05) (Table S4). Fourth, when restricted to 3,543 participants who continued to receive prescriptions for SPL or ESAX between three and six months, SPL users showed similar trends in insomnia risk (HR, 1.62; 95% CI, 1.23-2.15) (Table S5). Fifth, we additionally excluded 202 individuals with a history of sleep disorders. In this analysis, SPL initiation remained significantly associated with a higher incidence of sleep disorders (HR, 1.59; 95% CI, 1.26-2.00) (Table S6). Sixth, consistent results were observed in the analysis using multiple imputation for smoking status and alcohol consumption (HR, 1.69; 95% CI, 1.34-2.13) (Table S7).

## Discussion

From a nationwide database of health checkups and insurance claims, the present study identified individuals who newly started either SPL or ESAX. IPTW-based analyses revealed an increased incidence of insomnia among SPL users compared with those prescribed ESAX. These findings were consistently supported by several sensitivity analyses. To our knowledge, this is the first clinical investigation to implicate SPL use in the development of insomnia.

MRAs are established antihypertensive medications that offer effective blood pressure reductions, with notable benefits in resistant hypertension and primary aldosteronism ^(2), (3), (4)^. Furthermore, numerous studies have indicated their efficacy in heart failure and chronic kidney disease associated with diabetes ^(5), (6), (7), (8), (30)^. Nonetheless, concerns about adverse effects persist, which could help explain the underuse of MRAs, even in eligible patients ^(31)^. In addition to class-related risks such as hyperkalemia, SPL―the first-generation MRA―can cause hormonal side effects through its off-target activity at androgen and progesterone receptors. While the full impact has yet to be elucidated, gynecomastia is among the most recognized of these, occurring in approximately 10% of men during a mean follow-up of 24 months ^(5)^. This condition can be physically painful and psychologically distressing, possibly leading to sleep disturbances ^(32)^. Of note, there is an interrelation between testosterone levels and sleep health: while restricted sleep lowers morning testosterone, replacement therapy improves sleep conditions in men with hypogonadism ^(14), (16), (33)^. Late-onset hypogonadism, an age-related androgen deficiency affecting approximately 20% of men over age 70, often presents with insomnia ^(17), (18)^. These observations raise the possibility that SPL may play a role in the onset of insomnia, particularly in middle-aged and older men, highlighting a gap in our current understanding.

Using a nationally representative real-world database, we compared the risk of incident insomnia associated with SPL relative to ESAX. This next-generation, highly selective MRA has antihypertensive efficacy broadly comparable to SPL ^(22), (34), (35)^. Our analysis of approximately 5,000 individuals who were new users of either MRA revealed that SPL was significantly linked to the subsequent development of insomnia. This relationship generally held true across a range of subgroup analyses. Interestingly, sex-based subgroup analyses showed a consistent trend not only in men but also in women. In postmenopausal women, aside from its role in sexual desire, the effects of testosterone remain poorly defined ^(13)^. It is possible that testosterone also has a meaningful influence on sleep in this population. Of note, the increased risk of insomnia associated with SPL was observed in older individuals with markedly reduced sex hormone secretion; its diuretic effect, although relatively mild, might contribute to insomnia via nocturia ^(36)^. Another explanation could tentatively involve differences between SPL and ESAX in distribution to the central nervous system or anti-inflammatory and anti-fibrotic properties ^(23), (37), (38), (39)^. Regardless, the precise mechanisms linking SPL to insomnia are likely multifactorial and remain beyond the scope of this study, warranting further investigation.

The present study has important clinical implications. Sleep-related disturbances increase with age and are also prevalent among individuals with hypertension ^(20)^. In view of the association between sleep disorders and unfavorable outcomes―including elevated blood pressure, as well as reduced quality of life and increased mortality―it appears reasonable to prioritize newer MRAs with presumed improvements in safety and tolerability ^(20), (21)^. Nonetheless, SPL remains an appropriate option depending on kidney function or drug availability. In routine clinical care, individuals do not tend to report insomnia symptoms spontaneously, and clinicians often do not inquire proactively about sleep, leading to underdetection of sleep disorders ^(40), (41)^. When prescribing SPL, clinicians should pay close attention to the emergence of insomnia.

A key strength of this study lies in the use of a large-scale, comprehensive nationwide dataset. This framework, combined with IPTW analyses adjusting for numerous confounders, enabled us to demonstrate that SPL is associated with a higher risk of insomnia compared with ESAX. However, several limitations should also be acknowledged. First, as a retrospective cohort study, this research could not entirely rule out residual confounding. It is important to note that we were unable to adequately adjust for socioeconomic status, although the sensitivity analysis incorporating the urbanity of the insurer location yielded results consistent with the main findings. While ESAX is moderately priced and widely used in Japan, it is more expensive than SPL, which is available as a generic formulation. Accordingly, this limitation should still be taken into account when interpreting the results. Second, since insomnia was defined using ICD-10 codes, there may be concerns about diagnostic accuracy. On the other hand, the incidence observed in this study was broadly consistent with previous reports (approximately 3%-30% per year, depending on the definition and cohort) ^(42), (43), (44), (45), (46)^. Moreover, it has been reported that ICD criteria may underestimate the burden of insomnia, suggesting that any misclassification bias is unlikely to have materially overstated the association between SPL use and insomnia ^(47)^. Third, this study relied on prescription data, and medication adherence could not be assessed, which represents a notable concern. Fourth, it should be noted that the stabilized IPTW approach may reduce the effective sample size. Finally, the cohort was almost exclusively drawn from the Japanese population, limiting the generalizability of the findings to other ethnic groups.

In conclusion, we found that, among individuals with hypertension, SPL use was significantly associated with an increased likelihood of developing insomnia compared to ESAX. Considering its widespread global use, this potential risk should be recognized by all medical professionals―not only prescribing clinicians―and addressed appropriately in clinical practice.

## Article Information

### Author Contributions

Hidehiro Kaneko and Takashin Nakayama conceived and designed the study. Hidehiro Kaneko, Takashin Nakayama, Akira Okada, Yuta Suzuki, Hiroyuki Morita, and Norifumi Takeda drafted the manuscript. Takashin Nakayama, Akira Okada, Yuta Suzuki, and Hideo Yasunaga performed the statistical analysis.

Hidehiro Kaneko, Takashin Nakayama, Akira Okada, Yuta Suzuki, Hiroyuki Morita, Katsuhito Fujiu, Norifumi Takeda, Tatsuhiko Azegami, Hideo Yasunaga, and Kaori Hayashi contributed to data interpretation. Katsuhito Fujiu, Norihiko Takeda, Takashi Yokoo, Koichi Node, Hideo Yasunaga, and Kaori Hayashi critically revised the manuscript for important intellectual content. All authors approved the final version of the manuscript and agree to be accountable for all aspects of the work.

### Conflicts of Interest

Research funding and scholarship funds (Hidehiro Kaneko and Katsuhito Fujiu) from Medtronic Japan Co., LTD, Boston Scientific Japan Co., LTD, BIOTRONIK JAPAN INC, SIMPLEX QUANTUM Inc, and UT-Heart Inc. Hidehiro Kaneko holds shares in PrevMed Co., Ltd. and Japan Preventive Medicine Development Institute Co., Ltd. Hidehiro Kaneko has received lecture fees from Daiichi Sankyo Co., Ltd. The remaining authors declare that they have no relevant financial interests.

### IRB Approval Code and Name of the Institution

The Ethics Committee of the University of Tokyo reviewed and approved the study protocol (approval ID: 2021010NI).

## Supplement

Supplementary Material
