# Supplementary material for: Comparison of Insomnia Risk between Spironolactone and Esaxerenone in Individuals with Hypertension
Source: JMA J. 2026 May 29;9(5):1187–95. doi: 10.31662/jmaj.2025-0554 (PMC13639456; doi:10.31662/jmaj.2025-0554)
Supplement: Supplementary file 1 [file 2433-3298-9-5-1187-s001.pdf]

Supplementary Table 1. Baseline Characteristics of the Weighted Study Population

	ESAX users	SPL users	SMD
Age, years	71	71	-0.038
Male	0.55	0.55	0.006
Year of MRA initiation			
2019-2021	51	52	-0.005
2022-2024	49	48	0.005
Cigarette smoking			
Non-current	61	61	0.006
Current	12	12	-0.002
Unknown	28	28	-0.005
Alcohol consumption			
Non-daily	45	45	0.002
Daily	23	23	0.001
Unknown	32	32	-0.004
Body mass index, kg/m^2	24.7	24.7	0.007
Systolic blood pressure, mmHg	143	143	0.030
Diastolic blood pressure, mmHg	80	79	0.073
Comorbidities			
Diabetes mellitus	0.21	0.21	-0.007
Dyslipidemia	0.68	0.68	-0.001
Primary aldosteronism	0.04	0.04	0.001
Myocardial infarction	0.02	0.02	-0.005
Stroke	0.11	0.11	-0.001

Malignancy	0.14	0.14	0.014
Gastroesophageal reflux disease	0.38	0.38	-0.011
Thyroid gland disorders	0.08	0.08	-0.003
Sleep apnea	0.03	0.03	-0.027
Overactive bladder	0.11	0.12	-0.010
Depressive disorder	0.03	0.04	-0.013
Alzheimer's disease	0.03	0.03	0.003
Schizophrenia	0.01	0.01	-0.001
Medications			
Calcium channel blockers	0.62	0.62	0.005
RAS inhibitors	0.78	0.78	-0.005
Thiazide-type diuretics	0.23	0.22	0.013
Beta blockers	0.16	0.16	0.006
SGLT2 inhibitors	0.07	0.07	0.001
Systemic glucocorticoids	0.10	0.10	0.005
Topical glucocorticoids	0.15	0.15	-0.009
Anxiolytics	0.07	0.07	0.001
Analgesics	0.21	0.21	0.002
Laboratory data			
LDL–C, mg/dl	116	116	-0.005
HDL–C, mg/dl	61	61	-0.006
Triglycerides, mg/dl	129	129	-0.008
Hemoglobin A1c, %	5.9	5.9	-0.006
eGFR, ml/min/1.73 m^2	67	67	0.025

Abbreviations: eGFR = estimated glomerular filtration rate; ESAX = esaxerenone; HDL–C = high-density lipoprotein cholesterol; LDL–C = low-density lipoprotein cholesterol; MRA = mineralocorticoid receptor antagonist; RAS = renin–angiotensin system; SGLT2 = sodium–glucose cotransporter 2; SMD = standardized mean difference; SPL = spironolactone.

Supplementary Table 2. Risk of Insomnia Among SPL and ESAX Users: Overlap Weighting Analysis

	No. of participants	No. of events	IR (95%CI)	Crude HR (95%CI)	Weighted HR (95%CI)
SPL users	1,910	207	6.47 (5.65–7.41)	2.18 (1.77–2.70)	1.77 (1.41–2.23)
ESAX users	3,181	149	3.00 (2.55–3.52)	1 [Reference]	1 [Reference]

IRs were calculated per 100 person-years. Weighted HRs were estimated using Cox proportional hazards models with overlap weighting. The models accounted for age, sex, mineralocorticoid receptor antagonist initiation period, initiation period, smoking status, alcohol consumption, body mass index, systolic blood pressure, diabetes mellitus, dyslipidemia, primary aldosteronism, myocardial infarction, stroke, malignancy, depression, Alzheimer's disease, schizophrenia, overactive bladder, sleep apnea syndrome, gastroesophageal reflux disease, thyroid gland disorders, use of calcium channel blockers, renin–angiotensin system inhibitors, thiazide-type diuretics, beta-blockers, sodium–glucose cotransporter 2 inhibitors, anxiolytics, analgesics, systemic glucocorticoids, and topical glucocorticoids, and estimated glomerular filtration rate.
Abbreviations: CI = confidence interval; ESAX = esaxerenone; HR = hazard ratio; IR = incidence rate; SPL = spironolactone.

Supplementary Table 3. Risk of Insomnia Among SPL and ESAX Users: Inverse Probability of Treatment Weighting Analysis (Additional Adjustment for the urban status of the insurer's location)

	No. of participants	No. of events	IR (95%CI)	Crude HR (95%CI)	Weighted HR (95%CI)
SPL users	1,802	198	6.67 (5.80–7.67)	2.09 (1.68–2.59)	1.64 (1.29–2.09)
ESAX users	2,822	139	3.23 (2.73–3.81)	1 [Reference]	1 [Reference]

IRs were calculated per 100 person-years. Weighted HRs were estimated using Cox proportional hazards models with inverse probability of treatment weighting. The models accounted for age, sex, mineralocorticoid receptor antagonist initiation period, initiation period, urban status of the insurer's location, smoking status, alcohol consumption, body mass index, systolic blood pressure, diabetes mellitus, dyslipidemia, primary aldosteronism, myocardial infarction, stroke, malignancy, depression, Alzheimer's disease, schizophrenia, overactive bladder, sleep apnea syndrome, gastroesophageal reflux disease, thyroid gland disorders, use of calcium channel blockers, renin–angiotensin system inhibitors, thiazide-type diuretics, beta-blockers, sodium–glucose cotransporter 2 inhibitors, anxiolytics, analgesics, systemic glucocorticoids, and topical glucocorticoids, and estimated glomerular filtration rate. Abbreviations: CI = confidence interval; ESAX = esaxerenone; HR = hazard ratio; IR = incidence rate; SPL = spironolactone.

Supplementary Table 4. Risk of Insomnia Among SPL and ESAX Users: Inverse Probability of Treatment Weighting Analysis (Restricted to Individuals Receiving Calcium Channel Blockers, Renin–Angiotensin System Inhibitors, or Thiazide-Type Diuretics)

	No. of participants	No. of events	IR (95%CI)	Crude HR (95%CI)	Weighted HR (95%CI)
SPL users	1,713	189	6.55 (5.68–7.55)	2.12 (1.71–2.63)	1.62 (1.28–2.05)
ESAX users	3,003	147	3.14 (2.67–3.69)	1 [Reference]	1 [Reference]

IRs were calculated per 100 person-years. Weighted HRs were estimated using Cox proportional hazards models with inverse probability of treatment weighting. The models accounted for age, sex, mineralocorticoid receptor antagonist initiation period, initiation period, smoking status, alcohol consumption, body mass index, systolic blood pressure, diabetes mellitus, dyslipidemia, primary aldosteronism, myocardial infarction, stroke, malignancy, depression, Alzheimer's disease, schizophrenia, overactive bladder, sleep apnea syndrome, gastroesophageal reflux disease, thyroid gland disorders, use of calcium channel blockers, renin–angiotensin system inhibitors, thiazide-type diuretics, beta-blockers, sodium–glucose cotransporter 2 inhibitors, anxiolytics, analgesics, systemic glucocorticoids, and topical glucocorticoids, and estimated glomerular filtration rate. Abbreviations: CI = confidence interval; ESAX = esaxerenone; HR = hazard ratio; IR = incidence rate; SPL = spironolactone.

Supplementary Table 5. Risk of Insomnia Among SPL and ESAX Users: Inverse Probability of Treatment Weighting Analysis (restricted to individuals with continued prescriptions between 3 and 6 months)

	No. of participants	No. of events	IR (95%CI)	Crude HR (95%CI)	Weighted HR (95%CI)
SPL users	1,136	114	5.33 (4.43–6.40)	2.03 (1.56–2.63)	1.62 (1.23–2.15)
ESAX users	2,407	111	2.68 (2.22–3.23)	1 [Reference]	1 [Reference]

IRs were calculated per 100 person-years. Weighted HRs were estimated using Cox proportional hazards models with inverse probability of treatment weighting. The models accounted for age, sex, mineralocorticoid receptor antagonist initiation period, initiation period, smoking status, alcohol consumption, body mass index, systolic blood pressure, diabetes mellitus, dyslipidemia, primary aldosteronism, myocardial infarction, stroke, malignancy, depression, Alzheimer's disease, schizophrenia, overactive bladder, sleep apnea syndrome, gastroesophageal reflux disease, thyroid gland disorders, use of calcium channel blockers, renin–angiotensin system inhibitors, thiazide-type diuretics, beta-blockers, sodium–glucose cotransporter 2 inhibitors, anxiolytics, analgesics, systemic glucocorticoids, and topical glucocorticoids, and estimated glomerular filtration rate. Abbreviations: CI = confidence interval; ESAX = esaxerenone; HR = hazard ratio; IR = incidence rate; SPL = spironolactone.

Supplementary Table 6. Risk of Sleep Disorders Among SPL and ESAX Users: Inverse Probability of Treatment Weighting Analysis

	No. of participants	No. of events	IR (95%CI)	Crude HR (95%CI)	Weighted HR (95%CI)
SPL users	1,848	204	6.59 (5.75–7.56)	1.90 (1.55–2.33)	1.59 (1.26–2.00)
ESAX users	3,041	167	3.54 (3.04–4.12)	1 [Reference]	1 [Reference]

IRs were calculated per 100 person-years. Weighted HRs were estimated using Cox proportional hazards models with inverse probability of treatment weighting. The models accounted for age, sex, mineralocorticoid receptor antagonist initiation period, initiation period, smoking status, alcohol consumption, body mass index, systolic blood pressure, diabetes mellitus, dyslipidemia, primary aldosteronism, myocardial infarction, stroke, malignancy, depression, Alzheimer's disease, schizophrenia, overactive bladder, sleep apnea syndrome, gastroesophageal reflux disease, thyroid gland disorders, use of calcium channel blockers, renin–angiotensin system inhibitors, thiazide-type diuretics, beta-blockers, sodium–glucose cotransporter 2 inhibitors, anxiolytics, analgesics, systemic glucocorticoids, and topical glucocorticoids, and estimated glomerular filtration rate. Abbreviations: CI = confidence interval; ESAX = esaxerenone; HR = hazard ratio; IR = incidence rate; SPL = spironolactone.

Supplementary Table 7. Risk of Insomnia Among SPL and ESAX Users: Inverse Probability of Treatment Weighting Analysis (Multiple Imputation for Missing Covariates)

	No. of participants	No. of events	IR (95%CI)	Crude HR (95%CI)	Weighted HR (95%CI)
SPL users	1,910	207	6.47 (5.65–7.41)	2.18 (1.77–2.70)	1.69 (1.34–2.13)
ESAX users	3,181	149	3.00 (2.55–3.52)	1 [Reference]	1 [Reference]

IRs were calculated per 100 person-years. Weighted HRs were estimated using Cox proportional hazards models with inverse probability of treatment weighting. The models accounted for age, sex, mineralocorticoid receptor antagonist initiation period, initiation period, smoking status, alcohol consumption, body mass index, systolic blood pressure, diabetes mellitus, dyslipidemia, primary aldosteronism, myocardial infarction, stroke, malignancy, depression, Alzheimer's disease, schizophrenia, overactive bladder, sleep apnea syndrome, gastroesophageal reflux disease, thyroid gland disorders, use of calcium channel blockers, renin–angiotensin system inhibitors, thiazide-type diuretics, beta-blockers, sodium–glucose cotransporter 2 inhibitors, anxiolytics, analgesics, systemic glucocorticoids, and topical glucocorticoids, and estimated glomerular filtration rate. We performed multiple imputations for missing smoking status and alcohol consumption. Abbreviations: CI = confidence interval; ESAX = esaxerenone; HR = hazard ratio; IR = incidence rate; SPL = spironolactone.
